# Pharmacological Characterization of 4-Methylthioamphetamine Derivatives

**DOI:** 10.3390/molecules25225310

**Published:** 2020-11-13

**Authors:** Fabrizzio G. Guajardo, Victoria B. Velásquez, Daniela Raby, Gabriel Núñez-Vivanco, Patricio Iturriaga-Vásquez, Rodrigo A. España, Miguel Reyes-Parada, Ramón Sotomayor-Zárate

**Affiliations:** 1 Laboratorio de Neuroquímica y Neurofarmacología, Centro de Neurobiología y Fisiopatología Integrativa (CENFI), Instituto de Fisiología, Facultad de Ciencias, Universidad de Valparaíso, Valparaíso 2360102, Chile; fabrizzioguajardo@live.cl (F.G.G.); victoria.velasquezp@gmail.com (V.B.V.); daniraby92@gmail.com (D.R.); 2 Center for Bioinformatics, Simulations and Modelling, University of Talca, Talca 3460000, Chile; ganunez@utalca.cl; 3 Laboratorio de Síntesis Orgánica y Farmacología Molecular, Departamento de Ciencias Químicas y Recursos Naturales, Facultad de Ingeniería y Ciencias, Universidad de la Frontera, Temuco 4811230, Chile; patricio.iturriaga@ufrontera.cl; 4 Center of Excellence in Biotechnology Research Applied to the Environment, Universidad de La Frontera, Temuco 4811230, Chile; 5 Department of Neurobiology and Anatomy, Drexel University College of Medicine, Philadelphia, PA 19129, USA; rae39@drexel.edu; 6 Centro de Investigación Biomédica y Aplicada (CIBAP), Escuela de Medicina, Facultad de Ciencias Médicas, Universidad de Santiago de Chile, Santiago 9170022, Chile; 7 Facultad de Ciencias de la Salud, Universidad Autónoma de Chile, Talca 3467987, Chile

**Keywords:** conditioned place preference (CPP), DAT, locomotor activity, reward, FSCV, molecular docking

## Abstract

Amphetamine derivatives have been used in a wide variety of pathologies because of their pharmacological properties as psychostimulants, entactogens, anorectics, and antidepressants. However, adverse cardiovascular effects (sympathomimetics) and substance abuse problems (psychotropic and hallucinogenic effects) have limited their use. 4-Methylthioamphetamine (MTA) is an amphetamine derivative that has shown to inhibit monoamine uptake and monoamine oxidase. However, the pharmacological characterization (neurochemical, behavioral, and safety) of its derivatives 4-ethylthioamphetamine (ETA) and 4-methylthio-phenil-2-butanamine (MT-But) have not been studied. In the current experiments, we show that ETA and MT-But do not increase locomotor activity and conditioned place preference with respect to MTA. At the neurochemical level, ETA and MT-But do not increase in vivo DA release in striatum, but ETA and MT-But affect the nucleus accumbens bioaccumulation of DA and DOPAC. Regarding cardiovascular effects, the administration of MTA and ETA increased the mean arterial pressure and only ETA significantly increases the heart rate. Our results show that the pharmacological and safety profiles of MTA are modulated by changing the methyl-thio group or the methyl group of the aminoethyl chain.

## 1. Introduction

Amphetamine is a drug with multiple pharmacological effects whose mechanism of action has not been fully elucidated. However, it is known that amphetamine and many of its derivatives bind to monoamine transporters, increasing the bioavailability of dopamine (DA), norepinephrine (NE), and/or serotonin (5-HT) in the synaptic cleft [1,2]. The pharmacological profile of amphetamine derivatives greatly depends on the number, type, and position of substituent(s) in the basic amphetamine structure [3,4,5]. Clinically, amphetamine and its derivatives have shown a wide therapeutic potential for the treatment of narcolepsy, obesity, and attention deficit hyperactive disorder (ADHD), among other conditions [1,6]. However, cardiovascular and neuropsychiatric side effects such as hypertension, tachycardia, valve disease, or addiction have limited their use in recent times. In this context, new amphetamine derivatives have been designed with the aim to maintain its psychostimulant properties while lessening cardiovascular side effects. For example, phentermine (α-methyl-amphetamine) is an approved drug to treat obesity with lower cardiovascular and dependence effects than amphetamine. On the other hand, lisdexamfetamine a pro-drug formed by the amino acid L-lysine attached to dextroamphetamine is used to treat ADHD [7] and binge-eating disorder [8], whereas ecstasy (3,4-methylenedioxymethamphetamine or MDMA) is an illegal drug used in rave parties which has been studied to treat patients with post-traumatic stress disorder [9,10].

**Figure 1:**
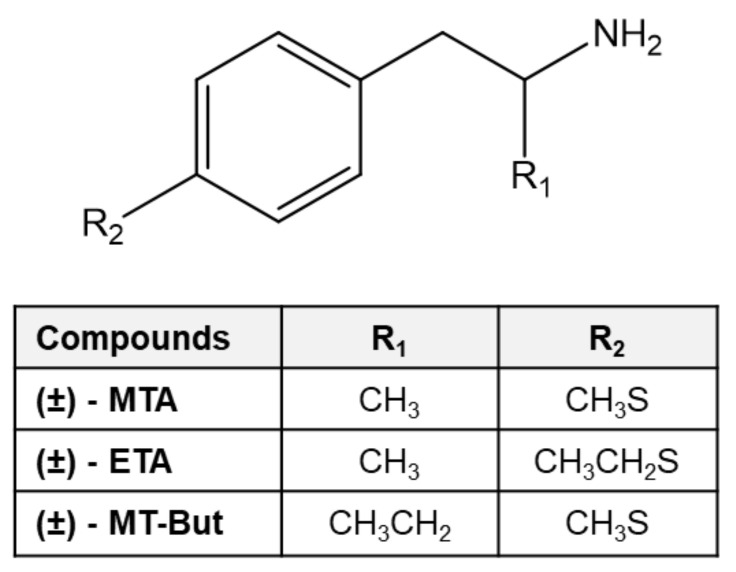
Chemical structure of 4-methylthioamphetamine (MTA) and its derivatives 4-ethylthioamphetamine (ETA) and 4-methylthio-phenil-2-butanamine (MT-But).

Another interesting amphetamine derivative is 4-methylthioamphetamine (MTA or commonly known as flatliner [11]. This drug increases extracellular levels of 5-HT and DA either through the blockade of the respective monoamine transporter or by inducing neurotransmitter efflux (or both) [12,13,14]. In addition, MTA is also a potent and selective monoamine oxidase type A (MAO-A) inhibitor [4,15,16]. Previously, our group has studied MTA and its derivative *N*,*N*-dimethyl-MTA, showing that both compounds increase brain extracellular levels of DA and 5-HT [17,18] and inhibit MAO-A activity [5,18,19]. Interestingly, *N*,*N*-dimethyl-MTA maintains its psychoactive activity, but does not produce in vitro aortic contractility [17], indicating a better safety profile than MTA. In this context, it seemed attractive to evaluate the neurochemical and behavioral effects of novel MTA derivatives such as 4-ethylthioamphetamine (ETA), which contains an ethylthio group at position 4 of the aromatic ring and 4-methylthio-phenil-2-butanamine (MT-But), which has an ethyl substitution at the alpha position of the ethylamine chain (Figure 1). Thus, in the present work we further characterized the dopaminergic effects of MTA and its derivatives ETA and MT-But. The actions of MTA, ETA, and MT-But upon DA transporter (DAT) were studied in silico and in vivo. Also, the possible abuse liability and cardiovascular safety profile of the drugs were assessed in vivo.

## 2. Results

### 2.1. Behavioral Data in Locomotor Activity and Conditioned Place Preference Tests

**Figure 2:**
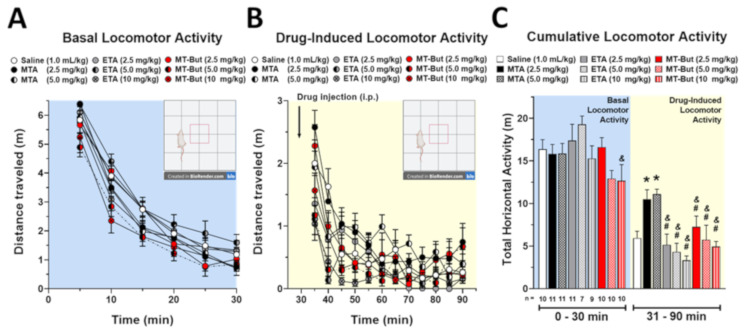
(**A**) Time course of basal locomotor activity expressed as distance traveled (m) from 0 to 30 min. (**B**) Time course of saline-induced and drug-induced locomotor activity (m) from 31 to 90 min. (**C**) Cumulative basal and induced locomotor activity (m) (* *P* < 0.05 Saline vs. all doses of MTA, ^#^ *P* < 0.05 MTA vs. all doses of ETA and ^&^ *P* < 0.05 vs. all doses of MT-But).

To examine the effects of amphetamine derivatives on locomotor activity, rats were treated with saline (1 mL/kg; *n* = 10), MTA (2.5 mg/kg; *n* = 11, 5.0 mg/kg; *n* = 11), ETA (2.5 mg/kg; *n* = 10, 5.0 mg/kg; *n* = 10, 10 mg/kg; *n* = 10) or MT-But (2.5 mg/kg; *n* = 11, 5.0 mg/kg; *n* = 7, 10 mg/Kg; *n* = 9). Treatments were administered via intraperitoneal (i.p.) injections after 30 min of basal locomotor activity were assessed (see Methods and Materials section). Figure 2A shows the temporal course of locomotor activity during 90 min for the experimental groups. Figure 2B shows basal (0–30 min) and post-injection (31–90 min) cumulative locomotor activity for all experimental groups. Basal cumulative locomotor activity was only different when ETA (5.0 mg/kg) vs. MT-But (10 mg/kg) [F_(8,80)_ = 2.084, *P* = 0.0469] is compared. The cumulative locomotor activity induced by MTA administration (31 to 90 min) was significantly higher than cumulative locomotor activity produced by saline, ETA, or MT-But injection [F_(8,80)_ = 7.451, *P* < 0.0001] (* *P* < 0.05 for Saline vs. MTA [2.5 and 5.0 mg/kg,], ^#,&^ *P* < 0.05 for MTA [2.5 and 5.0 mg/kg,] vs. all doses of ETA or MT-But. Neither ETA nor MT-But induced significant changes in cumulative locomotor activity as compared with control saline group.

**Figure 3:**
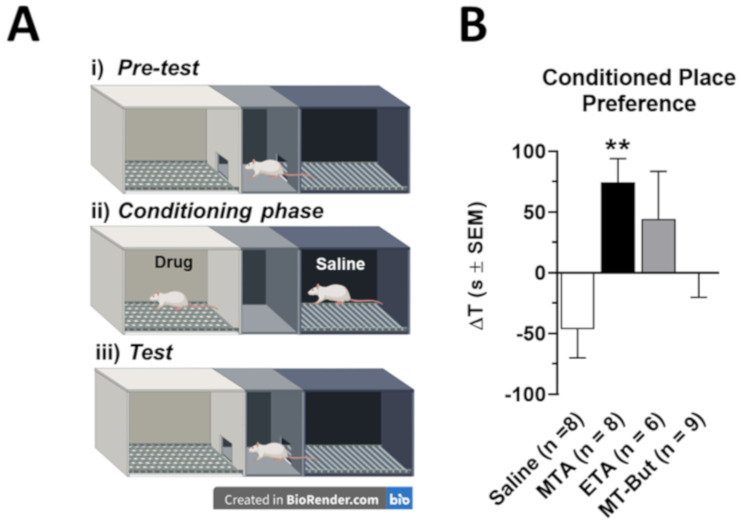
Conditioned place preference (CPP) to saline, MTA, ETA, or MT-But of adult male rats. (**A**) It shows a drawing of the CPP equipment and the phases used in this work. The drug administration was paired to the white compartment, while the saline administration to the black compartment. (**B**) Data are shown as the time difference (ΔT) in seconds between the time spent in the white compartment in the test phase and the pre-test phase (** *P* = 0.01).

Figure 3A shows a drawing of the CPP equipment used in this work. The administration of MTA (5.0 mg/kg, i.p.; *n* = 8), ETA (5.0 mg/kg, i.p.; *n* = 6) or MT-But (5.0 mg/kg, i.p.; *n* = 9) was paired to the white compartment, while saline administration (1 mL/kg, i.p.) was paired to the black compartment. In this context, Figure 3B shows that MTA administration induced a significant conditioned place preference with regard to the control group [F_(3,27)_ = 4.582, *P* = 0.0102] (** *P* = 0.01). By contrast, neither ETA nor MT-But elicited a significant preference for the white compartment, indicating that no conditioning preference was induced by these two drugs.

### 2.2. Neurochemical Data of Tissue Content and Release of DA

**Figure 4:**
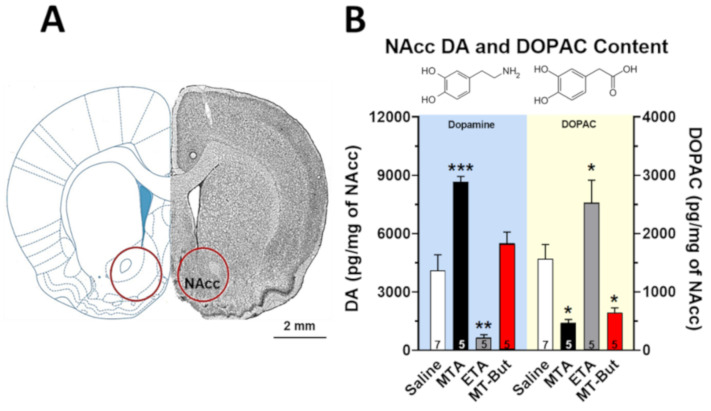
DA and 3,4-dihydroxyphenylacetic acid (DOPAC) content in NAcc of adult male rats. (**A**) Representative diagram of coronal section of rat brain. The red circles show the NAcc microdissected sections of 2 mm diameter obtained to analyze the DA and DOPAC content. (**B**) It shows the effects of the saline injections (1 mL/kg, i.p.; *n* = 7), MTA (5.0 mg/kg, i.p.; *n* = 5), ETA (5.0 mg/kg, i.p.; *n* = 5), or MT-But (5.0 mg/kg, i.p.; *n* = 5) on DA and DOPAC tissue content in NAcc of male rats (* *P* < 0.05; ** *P* < 0.01; *** *P* < 0.001 for saline vs. drugs).

**Figure 5:**
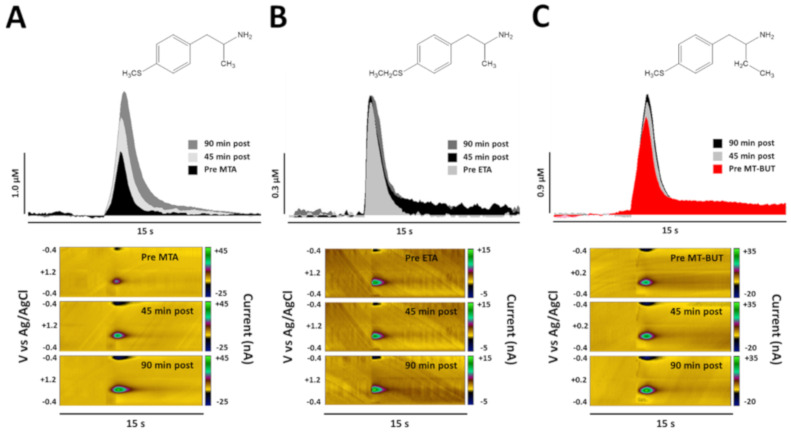
Representative peaks of DA oxidation and color plots obtained in basal condition and after 45- and 90-min post-injection of MTA (**A**), ETA (**B**), and MT-But (**C**) in dorsal striatum.

**Figure 6:**
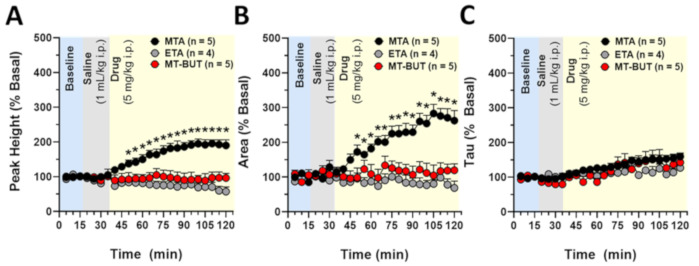
It shows the analysis regarding the baseline (%) for the peak height (**A**), area (**B**), and decay (**C**) of DA release in dorsal striatum evoked by electrical stimulation before and after MTA (5.0 mg/kg, i.p.; *n* = 5), ETA (5.0 mg/kg, i.p.; *n* = 4), and MT-But (5.0 mg/kg, i.p.; *n* = 5) administration (* *P* < 0.05 for drug comparisons).

Although it has been shown that the effects of psychostimulant drugs are different in magnitude at the level of NAcc and dorsal striatum [20], in this work we measured the tissue content of DA and DOPAC in NAcc (Figure 4), while DA release and uptake dynamics were determined with high temporal resolution in dorsal striatum (Figure 5 and Figure 6). Figure 4B shows the effects of saline (1 mL/kg, i.p.; *n* = 7), MTA (5.0 mg/kg, i.p.; *n* = 5), ETA (5.0 mg/kg, i.p.; *n* = 5) or MT-But (5.0 mg/kg, i.p.; *n* = 5) injections on DA and DOPAC tissue content in NAcc of male rats. Sixty minutes after injections, NAcc DA content is increased in the MTA group and reduced in the ETA group [F_(3,18)_ = 27.68, *P* < 0.0001] (** *P* < 0.01; *** *P* < 0.001). Furthermore, MT-But administration does not affect the NAcc DA content, although it tends to increase slightly. This slight effect of MT-But is also observed in Supplementary Figure S1 where NAcc extracellular levels of DA determined by in vivo brain microdialysis tend to increase 50 min post-drug administration. Regarding the NAcc DOPAC content, the main metabolite of DA in rodents, the administration of MTA and MT-But significantly reduce its level, while ETA increases it [F_(3,18)_ = 16.51, *P* < 0.0001] (* *P* < 0.05).

Figure 5 shows the representative peaks of DA overflow obtained pre- and post-drug administration (45 and 90 min) in dorsal striatum of anesthetized rats. Respective color plots are also shown, where DA concentration is plotted as green before and after injection of 5 mg/kg (i.p.) of MTA and its derivatives (ETA and MT-But).

Figure 6 shows the analysis regarding the baseline for the peak height (A), area (B), and decay (C) of DA release in dorsal striatum evoked by electrical stimulation before and after MTA (5.0 mg/kg, i.p.; *n* = 5), ETA (5.0 mg/kg, i.p.; *n* = 4), and MT-But (5.0 mg/kg, i.p.; *n* = 5) administration. Our data show that MTA increases peak height (interaction [F_(46,264)_ = 4.345, *P* < 0.0001]; time [F_(23,264)_ = 1.646, *P* = 0.0344; drug [F_(2,264)_ = 192.5, *P* < 0.0001]) and area under the curve (interaction [F_(46,264)_ = 4.217, *P* < 0.0001]; time [F_(23,264)_ = 3.885, *P* < 0.0001; drug [F_(2,264)_ = 167.0, *P* < 0.0001]) compared to ETA and MT-But administration. The comparisons for decay time (Tau) for MTA, ETA, and MT-But were only statistically different for time and drug variables (interaction [F_(46,264)_ = 0.6808, *P* = 0.9417]; time [F_(23,264)_ = 4.525, *P* < 0.0001; drug [F_(2,264)_ = 7.035, *P* = 0.0011]).

### 2.3. Cardiovascular Safety Profile

**Figure 7:**
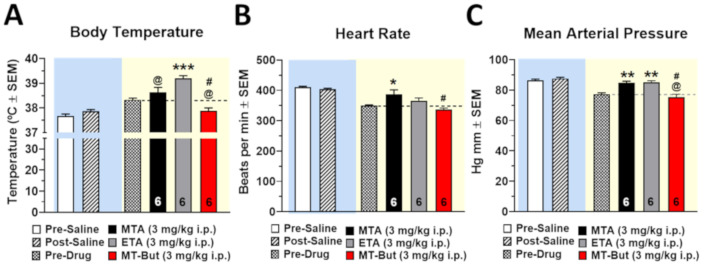
It shows the effects of MTA (5.0 mg/kg, i.p.; *n* = 6), ETA (5.0 mg/kg, i.p.; *n* = 6), and MT-But (5.0 mg/kg, i.p.; *n* = 6) administrations on body temperature (**A**), heart rate (**B**), and mean arterial pressure (**C**) (* *P* < 0.05; ** *P* < 0.01; *** *P* < 0.001 for drug administration vs. pre-drug; ^@^ *P* < 0.05 for MTA or MT-But vs. ETA; ^#^ *P* < 0.05 for MT-But vs. MTA).

Figure 7 shows the effects of MTA (3.0 mg/kg, i.p.; *n* = 6), ETA (3.0 mg/kg, i.p.; *n* = 6), and MT-But (3.0 mg/kg, i.p.; *n* = 6) on body temperature (A), heart rate (B), and mean arterial pressure (C) 15 min following administration of the drugs. Initially, the animals were anesthetized with chloral hydrate to obtain stable values in body temperature and cardiovascular parameters before saline administration (1.0 mL/kg, i.p.). Animals were injected with a second dose of anesthetic before drug administration, obtaining new stabilized values (pre-drug) of body temperature, heart rate, and mean arterial pressure. The choice of drug doses used in these experiments was based on previous studies of our lab where amphetamine was used as the positive control. In this context, the effects of drug administration on the body temperature were significantly different from the pre-drug values [F_(3,20)_ = 17.63, *P* < 0.0001]. Specifically, ETA increases body temperature relative to pre-drug levels (*** *P* < 0.001), and to MTA (^@^ *P* < 0.05) and MT-But (^@^ *P* < 0.05). Conversely, MT-But reduces body temperature relative to MTA administration (^#^ *P* < 0.05). Heart rate was only increased with MTA administration relative to pre-drug levels [F_(3,20)_ = 5.801, *P* = 0.0051] (* *P* < 0.05). Furthermore, MT-But only reduces heart rate with regard to MTA administration (^#^ *P* < 0.05). Mean arterial pressure was increased with MTA and ETA administration [F_(3,20)_ = 15.30, *P* < 0.0001] (** *P* < 0.01) relative to pre-drug levels. Finally, MT-But reduced mean arterial pressure relative to MTA and ETA (^@,#^ *P* < 0.05).

### 2.4. Molecular Docking of MTA, ETA, and MT-But on Rat (r)DAT Model

**Figure 8:**
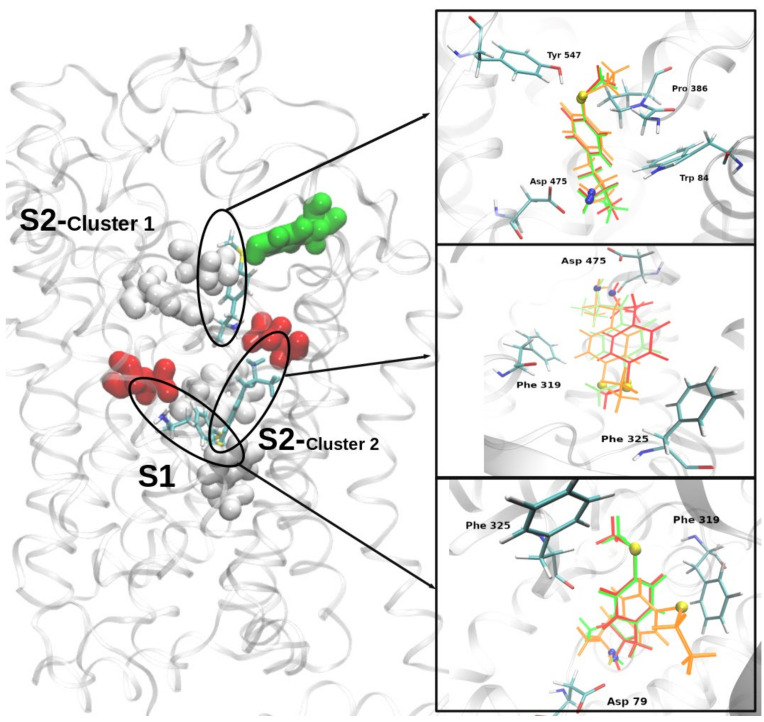
Left, ribbon diagram of the rDAT model generated showing the putative binding sites of MTA, ETA, and MT-But. Main active site aminoacid residues are shown in white/green (hydrophobic) or red (charged; ASP). Insets on the right show docking poses of MTA (red), ETA (orange), and MT-But (green) at S2 (cluster 1 up and cluster 2 middle) and S1 (bottom) sites. Main active sites residues (cyan) are rendered as stick models.

In an effort to gain insight regarding the molecular interactions underlying the differential effects of MTA, ETA, and MT-But upon DA levels, a homology model of rDAT was built and the three compounds were docked into it. A good quality model of rDAT was obtained after the modeling and molecular dynamics simulations processes (see RMSD behavior and quality evaluations on Supplementary Figures S2 and S3). Two independent docking experiments were performed for each ligand ((S)-MTA, (S)-ETA, and (S)-MT-But), using a box of 50 Å^3^ centered near to the residues Asp79 in the central binding site (the S1 site) or Asp475 in the extracellular vestibule site (the S2 site) [21,22,23]. After docking simulations, 50 conformers of each ligand were analyzed. Figure 8 shows that at both binding sites the three ligands exhibited very similar binding modes. In the case of the S1 site, all conformers were organized only in one cluster, whereas two clusters (with an RMSD larger than 3 Å between them) were obtained at the S2 site, which suggests that MTA, ETA, and MT-But might occupy two different locations into S2 site. At both binding sites, the three amphetamine derivatives appeared in a position in which their amino group (protonated) generates an electrostatic interaction with Asp residues (Asp79/S1, Asp475/S2), a binding mode which is further stabilized by a hydrogen bond established with residues Phe319 or Phe320 (at S1 and S2, respectively). In all cases, the aromatic ring of the amphetamine derivatives appears located in such a way as to establish π interactions with the aromatic groups of the residues forming the cavities (e.g., Phe319 and Phe325 at the S1 and S2 sites or Tyr547 at the S2 site). As shown in Table 1, the free energy of binding (Kcal/mol) calculated for the three ligands did not show significant differences neither between different ligands at the same site (e.g., MTA-S1 vs. ETA-S1), nor between the same ligand at the different sites (e.g., MTA-S1 vs. MTA-S2). Nevertheless, a slight preference seems to exist for the inner site (S1).

## 3. Discussion

In the present study we demonstrate that two MTA derivatives, ETA and MT-But, do not affect the striatal DA release and do not elicit the rewarding and locomotor effects that were previously showed for MTA [14]. These results are in agreement with data from the literature [24,25] showing that either the introduction of substituents on the aromatic ring of amphetamine or the extension of the side-chain α-methyl to an ethyl group significantly attenuates dopaminergic/psychostimulant activity. This contrasts with the structure–activity relationships observed for these derivatives in relation to their activity as MAO-A inhibitors, since both ETA and MT-But exhibit inhibitory activities in the same range of MTA [5]. Thus, our results agree with data indicating that the binding sites of DAT/MAO-A exhibit a low degree of structural similarity [26], and therefore subtle modifications can lead to compounds highly selective for each of the targets

### 3.1. Neurochemical Effects of MTA Derivatives

MTA increases extracellular DA levels in striatum (Figure 6) and NAcc (Figure S1). We have previously demonstrated using in silico, in vitro, and in vivo experiments that MTA is a DAT blocker [14]. In this work, we observed that MTA also increases NAcc DA tissue content at 60 min post-administration (Figure 4B), an effect that could be related to DAT inhibition, but also with the MAO-A inhibitory properties (IC_50_ 0.25 μM) of the compound [4,19]. This idea is supported by the significant reduction in NAcc DOPAC content (the main DA metabolite in rodent) induced by MTA (Figure 4B). Interestingly, even though ETA (IC_50_ 0.1 μM) [4] and MT-But (IC_50_ 0.84 μM) [19] also inhibit MAO-A with potencies in the same range of MTA, their effect upon NAcc DA tissue content were markedly different. Thus, MT-But also produces a reduction in NAcc DOPAC content, but it does not affect NAcc DA content (Figure 4B). These effects of MT-But may be related to a lower inhibitory activity on both DAT and MAO. Figure S1 shows that NAcc extracellular DA levels begin to increase after 1 h of MT-But administration, suggesting that the analysis of tissue content could require a longer time to observe a significant bioaccumulation of DA in NAcc. On the other hand, ETA administration elicited a neurochemical effect opposite to MTA in NAcc, that is, it produced a significant decrease in DA and an increase of DOPAC tissue content. This pharmacological profile of ETA is very interesting because it may imply neurochemical effects on others monoaminergic system such as serotonin (5-HT). In this context, mephedrone (4-methylmethcathinone) has been shown to affect DA and 5-HT systems, increasing DA and DOPAC content in NAcc and striatum and reducing DA and DOPAC in PFC [27]. Indeed, ETA showed a cardiovascular safety profile (Figure 7) similar to that of mephedrone, which produces an increase in arterial blood pressure and body temperature (among other effects) [28], both of which are coincident with symptoms of acute toxicity precipitated by serotonergic drugs. Nevertheless, further experiments are necessary to demonstrate the serotonergic effects of ETA and MT-But. Beyond these considerations, our results highlight/support the concept that very subtle changes of the amphetamine structure can markedly modify the effects of its derivatives.

Regarding DA release, we observed that ETA and MT-But did not increase striatal extracellular DA levels (Figure 6). Previously, we had demonstrated that systemic and intra-striatal MTA administration increases extracellular DA levels [14], which is dependent on its action as a DAT blocker. However, this is the first report that shows the real-time MTA effects on striatal DA level, total quantity and time decay using FSCV.

Another interesting finding of this work is associated with the structure–activity relationships observed for structural analogues of MTA. Previously, we have demonstrated that modifications such as dimethylation of the amino group in the aliphatic chain (i.e., *N*,*N*-dimethyl-MTA) maintains the pharmacodynamic properties of MTA on extracellular levels of DA and 5-HT [17,18]. However, the ethyl-thio substitution at position 4 of the aromatic group (ETA) or the ethyl substitution at the alpha position of the ethylamine chain (MT-But) leads to a suppression of the effects produced by MTA upon DA extracellular levels.

### 3.2. Behavioral Effects of MTA Derivatives

Our results show that ETA and MT-But administration do not affect cumulative locomotor activity or conditioned place preference (Figure 2 and Figure 3), as the parent compound has been shown to do. These behavioral data reflect the dopaminergic neurochemical profiles of MTA, ETA, and MT-But. Thus, MTA increases the content and extracellular DA levels in NAcc (Figure 4B and Figure S1) and striatum (Figure 5 and Figure 6), which should favor the synaptic transmission between dopaminergic neurons and medium spiny neurons [29], favoring the behavioral effects of the drug. Conversely, ETA and MT-But did not produce such behavioral effects likely because they do not affect DA striatal (dorsolateral and ventral) synaptic transmission.

### 3.3. In Vivo Safety Profile of MTA Derivatives

Body temperature significantly increased after ETA administration (Figure 7A), whereas no changes were observed after MTA or MT-But administration. Catecholaminergic molecules such as isoprenaline and epinephrine have been shown to produce a thermogenic effect through the activation of β_3_ adrenergic receptors in adipose tissue [30,31]. The activation of the signaling pathway of β_3_ adrenergic receptors favors the transcription of uncoupling protein-1 (UCP1) which leads to uncoupling of the electron transport chain in ATP synthesis [32,33]. This molecular pathway is responsible for the thermogenic effect mediated by catecholamines [34]. However, the hyperthermic effect produced by ETA could be generated by a serotonergic rather than a catecholaminergic effect, since as we observed (Figure 7B), ETA does not increase heart rate, an action that is mainly mediated by the activation of β_1_ adrenoceptors in the heart. On the other hand, it has been shown that MTA and *N*,*N*-dimethyl-MTA affect not only DA neurotransmission, but also increase extracellular levels of 5-HT [14,17,18]. In this context, although the serotonergic effect of ETA has not been determined, we cannot rule out that the hyperthermic effect of this drug could be mediated by the activation of the serotonergic system, as it is produced by other amphetamine derivatives such as 3,4-methylenedioxymethamphetamine [35].

Regarding heart rate, we observed that only administration of MTA increases it. This effect may be related to the previously demonstrated sympathomimetic effects of MTA [36] which seems to be related to an increase in extracellular levels of norepinephrine. Thus, structural modifications of the MTA molecule such as those in ETA, MT-But, or *N*,*N*-dimethyl-MTA [17] likely affect its activity upon norepinephrinergic neurotransmission, since we have shown (current and previous results) a loss of contractile activity on aortic rings and a lack of effect on heart rate.

### 3.4. In Silico Pharmacology of MTA, ETA, and MT-But on rDAT Model

With the aim to evaluate if the differential dopaminergic effects of MTA, ETA, and MT-But were associated with different abilities to interact with DAT, we conducted docking experiments on a homology model of the transporter. However, under the molecular simulation conditions used by us, MTA, ETA, and MT-But exhibited very similar binding modes (and theoretical affinities) at the two most commonly described DAT binding sites. This suggests that the distinct effects of the three derivatives are not related with their ability to bind the DAT, but presumably with differences in downstream interactions with the transporter protein. For example, differential effects upon the vesicular monoamine transporter-2 might explain the differing activities of the amphetamine derivatives evaluated here. Undoubtedly, further experiments are necessary to test this hypothesis. Furthermore, it is important to note that in addition to the most typical binding sites at S1 and S2, the three compounds appeared to bind to an alternative S2 subsite, which roughly coincides with a recently described novel cocaine-DAT binding site [37]. Further experiments are necessary to confirm if this site is relevant for the binding of amphetamine and its derivatives to the DAT.

## 4. Materials and Methods

### 4.1. Animals

Males Sprague-Dawley rats (60–80 days) were used in all experiments. The analyses were performed using 183 rats that showed a good state of health and were assigned to the following experimental groups: saline (*n* = 25), MTA (*n* = 50), ETA (*n* = 51), and MT-But (*n* = 57). All rats were kept under the same conditions of temperature (21 ± 2 °C), humidity (55 ± 5%), 12-h light-dark cycle (lights on at 08:00 h), food and water *ad libitum*. All experimental procedures were approved by the Bioethics and Biosafety Committee from the Universidad de Valparaíso, and the Institutional Animal Experimentation Ethics Board and the Science Council (FONDECYT) of Chile (N° BEA154-20 and N° BS002-20). Efforts were made to minimize the number of rats used and their suffering.

### 4.2. Reagents

MTA and MT-But (Figure 1) were synthesized as hydrochloride according to synthesis route previously published [19]. ETA was synthesized and kindly donated by Dr. David E. Nichols (Purdue University, U.S.A). Chemical structures of MTA, ETA and MT-But were confirmed by high-resolution one- and two-dimensional 1H- and 13C-NMR and purity was established by melting point and elemental analysis. DA and DOPAC standard, EDTA, and 1-octanesulfonic acid were purchased from Sigma-Aldrich, Inc. (St. Louis, MO, United States). All other reagents were of analytical and molecular grade.

### 4.3. Behavioral Studies

One hundred twenty rats were used for locomotor activity (*n* = 89) and CPP (*n* = 31) experiments.

#### 4.3.1. Locomotor Activity

Rats used for measuring the distance traveled were assigned to the following experimental groups: saline (*n* = 10), MTA (*n* = 22), ETA (*n* = 30) and MT-But (*n* = 27). The protocol used for locomotor activity experiments was previously described [38,39]. Briefly, rats were habituated to the test room for 1 h before starting the experimental protocol. Basal and drug-induced horizontal locomotor activity was measured using acrylic boxes (50 cm × 50 cm × 50 cm) (Figure 2A). Basal locomotor activity was registered for 30 min and then rats were injected with physiological saline solution (1 mL/kg, i.p.), MTA (2.5 and 5.0 mg/kg i.p.), ETA (2.5, 5.0 and 10 mg/kg i.p.), or MT-But (2.5, 5.0 and 10.0 mg/kg i.p.) followed by locomotor activity recording for 60 min. Each complete session of locomotor activity (90 min) was recorded with an IP camera (LX-C202 model; Lynx Security, Shanghai, China) and the total traveled distance (m) was analyzed using ANY-Maze software (Stoelting Co., Wood Dale, IL, United States). Test cages were wiped and cleaned with 5% ethanol solution between trials.

#### 4.3.2. Conditional Preference Place (CPP)

Rats used for CPP were assigned to the following experimental groups: saline (*n* = 8), MTA (*n* = 8), ETA (*n* = 6), and MT-But (*n* = 9). The CPP apparatus has two compartments of the same size (21 cm × 21 cm × 28 cm), white and black, and they are separated by a smaller central compartment with two guillotine doors (Figure 3A). This central compartment is gray and served as the start point during the place preference test. The white compartment had a smooth floor with white light of intensity 30 lux. The gray compartment had a smooth floor without illumination, while the black compartment had a mesh style floor without illumination. The CPP protocol consisted of three parts: pre-test (one day before the conditioning period), the conditioning period, and the test (24 h after the last injection). For the pre-test and the test, rats were placed in the neutral-gray center compartment with the two guillotine doors open and they could explore the entire apparatus for 15 min. The time spent in each compartment was measured by analyzing the recordings obtained by Internet protocol (IP) cameras (LX-C202 model; Lynx Security, Shanghai, China) fixed above each place preference apparatus and connected to a computer in another room. Rats showed a spontaneous preference for the black compartment during the pre-test session [14,17]. Therefore, we used a procedure in which the nonpreferred compartment (the white one) was associated with the reward induced by the drugs. The conditioning period lasted for 5 days and it started 24 h after pre-test. The non-preferred compartment (the white side) was associated with the reward induced by MTA, ETA, or MT-But (5 mg/kg i.p. per each drug) in the morning and they were confined to the white compartment for 60 min. In the afternoon, the same rats were confined to the black compartment after saline injection for 60 min. The saline group received the saline injection in the morning and afternoon in the black and white compartment, respectively. During the test phase, rats were placed in the central compartment of the apparatus with the two guillotine doors opened allowing free access to the compartments for 15 min. The time spent in each compartment was recorded and the time difference (ΔT) in seconds between the time spent in the white compartment on the test day and the pre-test day was used to determine the degree of conditioning in the rats.

### 4.4. Neurochemical Studies

Twenty-two rats were used for analyzing DA and DOPAC content in NAcc, and 14 rats were used for in vivo fast scan cyclic voltammetry experiments in dorsal striatum.

#### 4.4.1. NAcc DOPAC and DA Content Using HPLC Coupled to Electrochemical Detection

The animals were decapitated 60 min after injection of saline (1 mL/kg i.p.) or drugs (MTA, ETA or MT-But; 5 mg/kg i.p.). NAcc were bilaterally microdissected at 4 °C using a brain matrix (model 68711, RWD Life Science, Shenzhen, P.R. China) and micro-punch (model 15076; diameter 2.0 mm, Harris Uni-Core, Ted-Pella Inc., Redding, CA, USA) as described previously [40] (Figure 4A). Briefly, NAcc were weighed on an analytical balance (model JK-180, Chyo balance corp, Tokyo, Japan) and homogenized in 400 µL of 0.2 M perchloric acid using sonicator (model XL2005, Microson Ultrasonic Cell Disruptor, Heat Systems, Farmingdale, NY, USA). The homogenate was centrifuged to 12,000× *g* for 10 min at 4 °C (model Z233MK-2, Hermle Labor Technik GmbH, Wehingen, Germany) and the supernatant was filtered (model EW-32816-26; 0.2 µm, HPLC Syringe Filters PTFE, Cole-Parmer, Instrument Company, Vernon Hills, IL, USA) and the final supernatant was injected to HPLC-coupled electrochemical detector (ED). The concentration of DA and DOPAC was expressed as pg per mg of wet tissue.

#### 4.4.2. DA and DOPAC Quantification

Ten microliters of supernatant were injected to the HPLC-ED system with the following equipment: An isocratic pump, (model PU-2080 Plus, Jasco Co. Ltd., Tokyo, Japan), a C18 column (model Kromasil 100-3.5-C18, AkzoNobel, Bohus, Sweden), and an electrochemical detector (set at 650 mV, 0.5 nA; model LC-4C, BAS, West Lafayette, IN, United States). The mobile phase, containing 0.1 M NaH_2_PO_4_, 1.0 mM 1-octanesulfonic acid, 1.0 mM EDTA, and 8.0% (*v*/*v*) CH_3_CN (pH 3.4) was pumped at a flow rate of 0.125 mL/min. DA and DOPAC levels were assessed by comparing the respective peak area and elution time of the sample with a reference standard and the quantification was performed using a calibration curve for each neurotransmitter (Program ChromPass, Jasco Co. Ltd., Tokyo, Japan).

#### 4.4.3. In Vivo Fast Scan Cyclic Voltammetry to Measure DA Extracellular Levels in Dorsal Striatum

Rats were deeply anesthetized with isoflurane (3% in 0.8 L/min air flow) in an induction chamber for 3 min and placed in a stereotaxic apparatus (model 68002, RWD Life Science Co. Ltd., Shenzhen, China) with a mask to maintain anesthesia for all the experiment (isoflurane 1.5% in 0.8 L/min air flow), using an animal anesthesia system (model 510, RWD Life Science Co. Ltd., Shenzhen, China). Body temperature was maintained at 37 °C with a water circulation system (model 68662, RWD Life Science Co. Ltd., Shenzhen, China). Rats were exposed to a craniotomy for the implantation of 3 electrodes. A glassy-carbon microelectrode (working electrode) was implanted in dorsal striatum using the coordinates from the Rat Brain Atlas [41] (dorsal striatum: 1.3 mm posterior, 2.5 mm lateral, and 4.0 mm ventral to brain surface) and an Ag/AgCl reference electrode was positioned in the contralateral cortex. The electrode potential was linearly scanned (−0.4 to 1.2 V and back to −0.4 V vs. Ag/AgCl) and cyclic voltammograms were assessed at the carbon fiber electrode every 100 ms with a scan rate of 400 V/s using a voltammeter/amperometer (model Chem-Clamp Potensiostat, Dagan Corporation, Minneapolis, MN, USA). A bipolar stimulating electrode (model MS 303/2A, Plastics one Inc., Roanoke, VA, USA) was implanted in the midbrain using the coordinates from the Rat Brain Atlas [41] (Regard to bregma: 5.2 mm posterior, 1.3 mm lateral, and 7.5 mm ventral to brain surface). Phasic DA release was stimulated with the following parameters: monophasic +, 60 pulses, 60 Hz, 4 ms, 300 μA (current stimulus isolator NL800A; Digitimer, Ltd., Hertfordshire, UK). For data collection, two National Instruments acquisition cards (NI-DAQ; PCI-6711 and PCI-6052e; National Instruments, Austin, TX, USA) were used to interface the potentiostat and stimulator with Demon Voltammetry and Analysis software (Wake Forest Health Sciences, Winston-Salem, NC, USA) [42]. Data collection began once electrical stimulation in the midbrain induced a suitable dopamine peak in the striatum. Phasic DA was stimulated every 5 min and three steady baseline collections were measured. After basal period, a saline injection (1 mL/kg, i.p.) was made and for other 15 min DA release was measured. Finally, rats received an injection of MTA, ETA, or MT-But (5 mg/kg i.p.) and phasic DA release was assessed for 90 min after drug administration. Data were analyzed with Demon Voltammetry and Analysis software using peak height, area and tau as parameters of DA concentration induced by electrical stimulation and uptake kinetics. After each experiment, working electrodes were calibrated using a CSF containing 1 μM dopamine.

### 4.5. In Vivo Assessment of Blood Pressure, Heart Rate, and Body Temperature

Eighteen rats were used to measure cardiovascular parameters such as blood pressure (BP), heart rate (HR), and body temperature using the CODA™ Monitor (CODA™ Monitor, Kent Scientific Corporation, Torrington, CT, USA). This system is a validated noninvasive blood pressure system [43], that uses a specialized volume pressure recording sensor, and measures blood volume changes through a noninvasive tail-cuff method. CODA™ Monitor was set to measure six cardiovascular parameters (systolic BP, diastolic BP, mean arterial pressure, HR, tail blood volume, and blood flow) and body temperature each minute in rats. Our experimental protocol used rats anesthetized with chloral hydrate (CH: 320 mg/kg i.p.) and placed in a heating blanket programmed at 37 °C. When rats were deeply anesthetized the cardiovascular parameters were measured before (pre-saline) and after the saline administration (post-saline: 1 mL/kg i.p.). Forty minutes after the first dose of anesthesia, a quarter of initial dose of CH was administered to keep the animal anesthetized. Regularly this new dose of CH reduces the values of cardiovascular parameters regarding pre-saline conditions. Therefore, after the second injection of anesthetic, we expect to stabilize cardiovascular parameters and body temperature (pre-drug) before injecting a dose of 3 mg/kg i.p. of MTA, ETA, or MT-But (post-drug). In addition, we evaluate the cardiovascular effects of amphetamine and adrenaline as control drugs (data not shown).

### 4.6. Homology Modeling and Molecular Simulation

The crystal structures of the human serotonin transporter and the dopamine transporter from *Drosophila melanogaster* (PDB Ids 6vrh and 4xp4, respectively) were used as templates to build a rat DAT (rDAT) homology model. Both structures show favorable sequence identity values (6rvh: 57%, 4xp4: 51%) and coverages higher than 0.85 with rDAT. All the modeling process was done with the MODELLER tool as previously described [14]. Then, the model built was embedded into a POPC membrane, solvated and ions were added creating an overall neutral system in approximately 0.02 M NaCl. To obtain an equilibrated structure of the model, the final system was subjected to a molecular dynamics (MD) simulation of 40 ns using the NAMD program (2.12 version, NIH, Chicago, IL, USA). The last frame of the simulation was used to extract the new coordinates of the model. This model was then geometrically, energetically, and stereochemically evaluated using PROSAII (Web-server version, University of Salzburg, Hellbrunnerstrasse, Austria) and Procheck tools (3.5.4 version, European Bioinformatics Institute, Cambridge, UK).

### 4.7. Molecular Docking

We used the AutoDock4.0 tool (San Diego, CA, USA) to estimate the free energy of binding of the ligands (S)-MTA, (S)-ETA, and (S)-MT-But on the structure of the generated rDAT model. The choice of the (S)-isomer for docking experiments was made on the basis that, in most cases, (S)-amphetamine derivatives (which are always dextrorotatory) are the eutomers at the DAT [24,44]. These three ligands, on their protonated form, were constructed and optimized using the program Gaussian03 (Cambridge, UK). For the docking simulations, two grid box were centered on the currently known bindings sites of DAT, whose names are S1 (inner site) and S2 (external site). In both cases the size of the box was 50 × 50 × 50, and the rest of the parameters were set as previously described [14].

### 4.8. Statistical Analysis

Data were expressed as mean ± SEM. One-way ANOVA followed by post-hoc Tukey test (multiple comparisons) was performed for behavioral (Figure 2B and Figure 3B), neurochemical (Figure 4B), and cardiovascular pharmacology (Figure 7) experiments. Two-way ANOVA followed by post-hoc Tukey test (multiple comparisons) was performed for the data analysis of in vivo fast scan cyclic voltammetry experiments (Figure 6). The statistical analyses were carried out with GraphPad Prism version 8.2.1 (GraphPad Software, San Diego, CA, United States) and *P* < 0.05 was considered statistically significant.

## 5. Conclusions

MTA is a ductile molecule that allows designing and synthesizing new derivatives that could exert different pharmacological effects on endogenous monoaminergic systems. In this sense, methyl substitutions in MTA molecule specifically in the amino group of the parent scaffold, maintain the dopaminergic and serotonergic activity of MTA. However, as shown in the present work, the replacement of the methyl-thio group at the aromatic ring by an ethyl-thio moiety, or the modification of the methyl group in the alpha carbon of the aminoethyl chain to an ethyl group (both with more steric hindrance than the original substituents), lead to a marked/complete decrease/loss of dopaminergic activity. Thus, our results indicate that the *para* position in the aromatic ring and/or the alpha position of the aminoethyl chain are key-determinants for the MTA effects on DA, and unveil a narrow structural window for alkyl substitution and the dopaminergic profile of the MTA derivatives. In this context, it seems very valuable to seek for novel pharmacological tools that, by showing an ample spectrum of efficacies, could be useful to treat neuropsychiatric and metabolic pathologies related to DA neurotransmission, such as ADHD and obesity, respectively.

## Figures and Tables

**Table 1:** The free energy of binding (Kcal/mol) calculated for the three ligands.

Ligand	Free Energy of Binding (Kcal/mol)		
	S1	S2	
		Cluster 1	Cluster 2
**(±)-MTA**	−6,4	−5,5	−5,3
**(±)-ETA**	−6,4	−5,5	−5,4
**(±)-MT-But**	−6,6	−5,3	−5,3

## Supplementary Materials

The following are available online, Figure S1: The baseline (0–30 min) and induced-drug (31–140 min) extracellular levels of DA in NAcc. MTA increases DA levels relative to their own baseline levels and relative to induced-MT-But DA levels. Figure S2: rDAT backbone RMSD variation during 20 ns of molecular dynamics. The time elapsed between each frame corresponds to 5ps. Figure S3: rDAT model evaluation confirms the quality of it. Black dot in PROSA plot (left) indicates that the model is in the adequate range according to the size structure. Ramachandran plot (right) shows that most of residues are in the most favored regions, while almost no residues were found in disallowed regions.
