# Supplementary material for: Pharmacological Characterization of 4-Methylthioamphetamine Derivatives
Source: Molecules. 2020 Nov 13;25(22):5310. doi: 10.3390/molecules25225310 (PMC7696343; doi:10.3390/molecules25225310)
Supplement: Supplementary file 1 [file molecules-25-05310-s001.pdf]

Supplementary Materials

Pharmacological Characterization of 4-Methylthioamphetamine Derivatives

Fabrizzio G. Guajardo[1,#], Victoria B. Velásquez[1,#], Daniela Raby[1], Gabriel Núñez-Vivanco[2], Patricio Iturriaga-Vásquez[3,4], Rodrigo A. España[5], Miguel Reyes-Parada[6,7,*] and Ramón Sotomayor-Zárate[1,*]

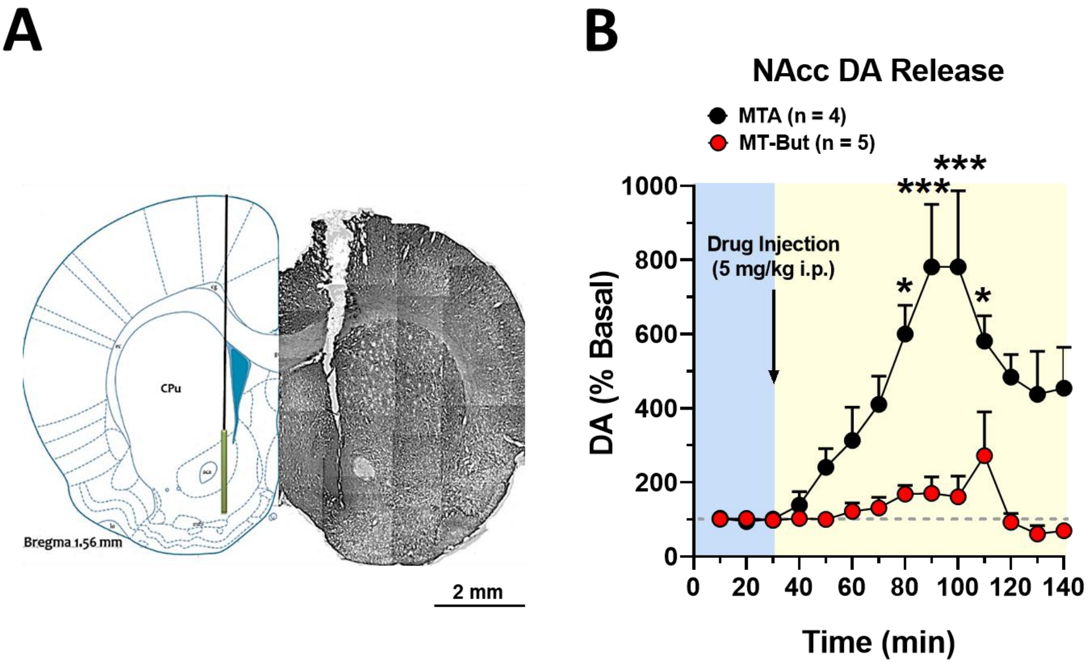

Figure S1. The baseline (0 - 30 min) and induced-drug (31 - 140 min) extracellular levels of DA in NAcc. MTA increases DA levels relative to their own baseline levels and relative to induced-MT-But DA levels.

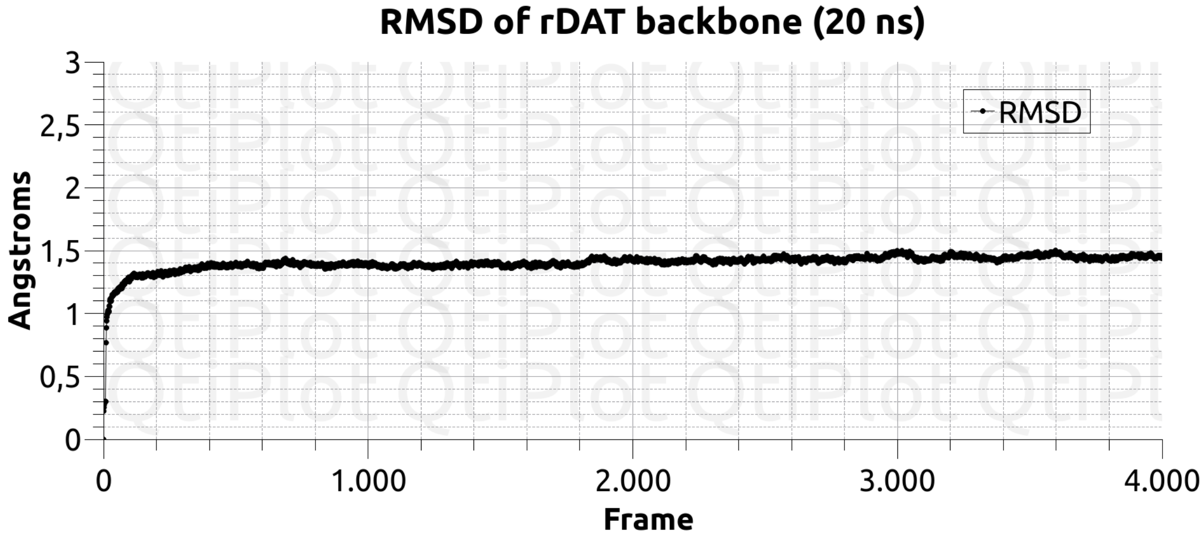

Figure S2. rDAT backbone RMSD variation during 20 ns of molecular dynamics. The time elapsed between each frame corresponds to 5ps.

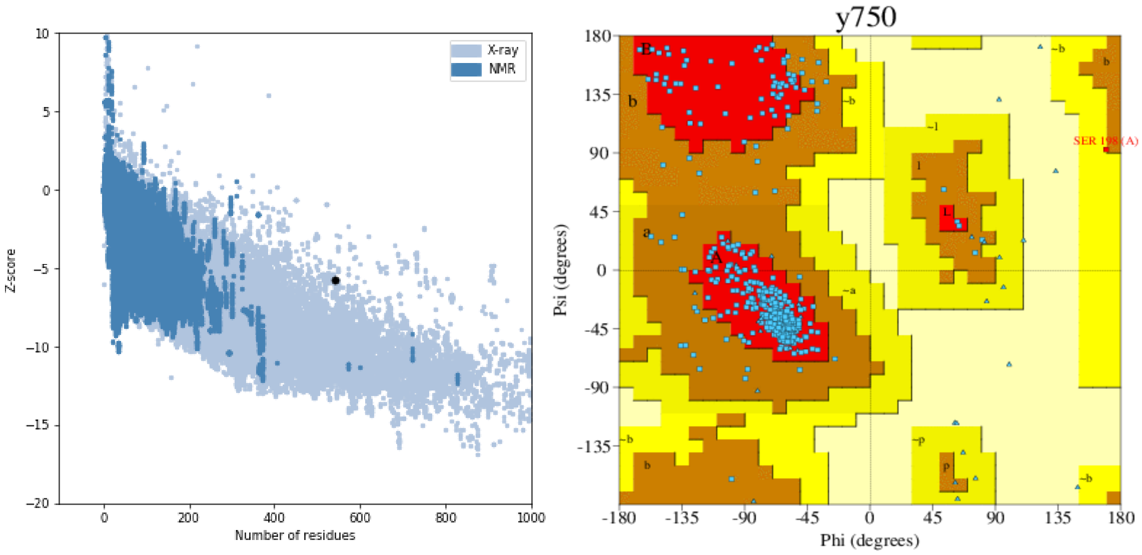

Figure S3. rDAT model evaluation confirms the quality of it. Black dot in PROSA plot (left) indicates that the model is in the adequate range according to the size structure. Ramachandran plot (right) shows that most of residues are in the most favored regions, while almost no residues were found in disallowed regions.
